# Biallelic *VPS41* Variants in Autosomal Recessive Spinocerebellar Ataxia 29 Resolved by Long‐Read Sequencing and RNA Analysis

**DOI:** 10.1002/mgg3.70285

**Published:** 2026-08-27

**Authors:** Natsuki Nakamura, Yosuke Nishio, Hiromi Nyuzuki, Ai Fukushima, Masaki Miura, Yu Kobayashi, Risako Ishioka, Kotaro Tsukada, Yasuyoshi Oka, Koyo Tsujikawa, Hironobu Morinaga, Mie Inaba, Jun Tohyama, Yuka Nakazawa, Takeshi Ikeuchi, Takeshi Ono, Shinji Saitoh, Tomoo Ogi

**Affiliations:** ^1^ Department of Pediatrics Central Hospital, Aichi Developmental Disability Center Kasugai Japan; ^2^ Department of Genetics, Research Institute of Environmental Medicine (RIeM) Nagoya University Nagoya Japan; ^3^ Department of Human Genetics and Molecular Biology Nagoya University Graduate School of Medicine Nagoya Japan; ^4^ Medical Genomics Center Nagoya University Hospital Nagoya Japan; ^5^ Department of Pediatrics Nagoya University Graduate School of Medicine Nagoya Japan; ^6^ Center for Medical Genetics Niigata University Medical and Dental Hospital Niigata Japan; ^7^ Niigata Prefecture Hamagumi Medical Rehabilitation Center for Disabled Children Niigata Japan; ^8^ Department of Child Neurology National Hospital Organization Nishiniigata Chuo Hospital Niigata Japan; ^9^ Department of Pediatrics and Neonatology Nagoya City University Graduate School of Medical Sciences Nagoya Japan; ^10^ Department of Molecular Genetics, Center for Neurological Diseases and Cancer Nagoya University Graduate School of Medicine Nagoya Japan; ^11^ Department of Pediatrics Niigata University Graduate School of Medical and Dental Sciences Niigata Japan

**Keywords:** haplotype phasing, long‐read sequencing, spinocerebellar ataxia 29, splicing, VPS41

## Abstract

**Background:**

Biallelic variants in *VPS41*, encoding a subunit of the HOPS complex, cause autosomal recessive spinocerebellar ataxia 29 (SCAR29), a rare neurodevelopmental disorder with an incompletely defined phenotypic and molecular spectrum.

**Methods:**

We investigated a 24‐year‐old man with cerebellar ataxia, hypotonia, and intellectual disability. Exome sequencing identified four candidate *VPS41* variants. Because maternal DNA was unavailable, long‐read genome sequencing was performed to determine allelic configuration, followed by RNA and protein analyses.

**Results:**

In addition to typical SCAR29 features, the patient showed previously unreported findings, including swan‐neck deformities and pes cavus. Long‐read genome sequencing demonstrated that two *VPS41* variants were in trans. RNA analysis revealed distinct splicing consequences: one allele produced an out‐of‐frame transcript predicted to undergo nonsense‐mediated decay, whereas the other generated an in‐frame exon‐skipped transcript. These complementary defects reduced *VPS41* expression at both transcript and protein levels, supporting pathogenicity and variant reclassification.

**Conclusion:**

Our findings expand the phenotypic spectrum of *VPS41*‐related disease and highlight the value of long‐read allelic resolution in clarifying pathogenic mechanisms in rare genetic disorders.

## Graphical abstract

Long‐read sequencing phased multiple VPS41 variants and established the biallelic configuration in a patient with autosomal recessive spinocerebellar ataxia 29. Transcript analysis revealed distinct allele‐specific splicing abnormalities, while the patient also showed clinical features beyond the typical phenotype.

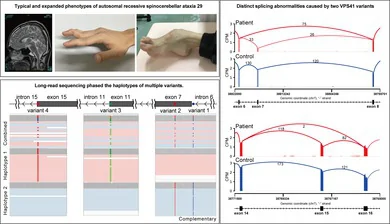

## 1 Introduction

*VPS41* encodes a subunit of the homotypic fusion and vacuole protein sorting (HOPS) complex, a hexameric tethering complex that mediates fusion of lysosomes with late endosomes and autophagosomes and plays a central role in autophagy and endolysosomal trafficking (van der Beek et al. 2024; van der Beek and Klumperman 2025). Disruption of this pathway has been implicated in a range of human diseases, including lysosomal storage disorders, neurodevelopmental syndromes, and neurodegenerative conditions (Marques and Saftig 2019; Lie and Nixon 2019; Healy et al. 2023; Ballabio and Bonifacino 2020).

Biallelic loss‐of‐function variants in *VPS41* have been reported to cause an autosomal recessive spinocerebellar ataxia (SCAR29) characterized by cerebellar ataxia, cognitive impairment, and dystonia (Monfrini et al. 2021; Sanderson et al. 2021; Steel et al. 2020; van der Welle et al. 2021). Although VPS41‐related disorders were first reported in 2020, fewer than 20 patients have been described to date, and the phenotypic spectrum remains incompletely defined. Here, we report a patient with biallelic *VPS41* variants who exhibited previously unreported features, including swan‐neck deformities and foot deformities. Long‐read sequencing enabled haplotype phasing when maternal DNA was unavailable, and functional analyses provided evidence supporting pathogenicity.

## 2 Results

### 2.1 Patient

The proband is a 24‐year‐old male with early‐onset cerebellar ataxia, hypotonia, and intellectual disability. He also developed pes cavus, anterior‐predominant cerebellar atrophy on brain MRI, and progressive swan‐neck deformities of the second to fourth fingers bilaterally. Cognitive assessment (WAIS‐IV) showed a full‐scale IQ of 49. Nerve conduction studies and spinal cord MRI (C1‐T6; age 10 years) were normal, and prior genetic testing, including a comprehensive NGS panel for Charcot–Marie–Tooth disease and inherited peripheral neuropathies (103 genes) and chromosomal microarray analysis, was non‐diagnostic. Further clinical and comparative details are provided in the Supporting Information, including Figure S1 and Table S1.

### 2.2 Genetic Analysis and Variant Prioritization

**FIGURE 1:**
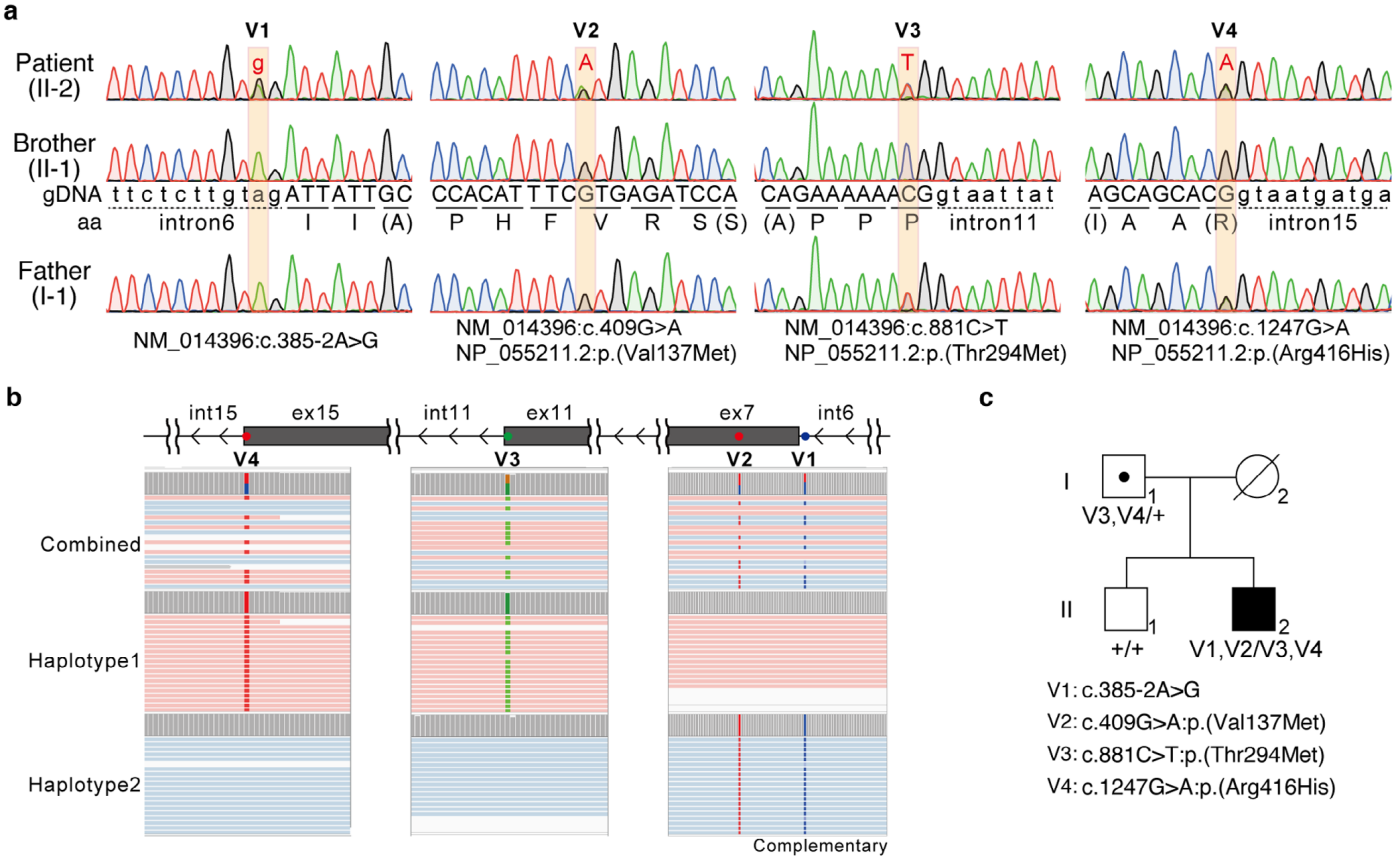
Genetic analysis of the patient. (a) Sanger sequencing results of the patient (II‐2), his elder brother (II‐1), and father (I‐1). Variant 1 (V1: NM_014396.4:c.385‐2A>G) and variant 2 (V2: NM_014396.4:c.409G>A (NP_055211.2:p.Val137Met)) were observed exclusively in the proband. In contrast, Variant 3 (V3: NM_014396.4:c.881C>T (NP_055211.2:p.Thr294Met)) and Variant 4 (V4: NM_014396.4:c.1247G>A (NP_055211.2:p.Arg416His)) were inherited from the father. The elder brother did not carry any of these variants. The light orange boxes indicate the positions of the base substitutions. (aa, amino acid; gDNA, genomic DNA). (b) Upper panel: Schematic representation of *VPS41* showing the positions of the four variants. Substituted bases are color‐coded: T (red), A (green), and C (blue). Lower panel: Integrative Genomics Viewer (IGV) visualization of sequencing reads around each variant, colored by haplotype. “Combined” shows raw alignment data including both haplotypes, whereas “Haplotype 1” and “Haplotype 2” show phased reads corresponding to each haplotype. ex, exon; int., intron. (c) Summary of genetic findings annotated on the family pedigree. Each haplotype is separated by a slash (/). “+” indicates the reference base.

Exome sequencing identified four *VPS41* variants confirmed by Sanger sequencing (Figure 1a): NM_014396.4:c.385‐2A>G (Variant 1, V1), NM_014396.4:c.409G>A (NP_055211.2:p.Val137Met; V2), NM_014396.4:c.881C>T (NP_055211.2:p.Thr294Met; V3), and NM_014396.4:c.1247G>A (NP_055211.2:p.Arg416His; V4), with no other pathogenic candidates detected. Segregation analysis revealed that V3 and V4 were inherited from the father, whereas the inheritance of V1 and V2 could not be determined because maternal DNA was unavailable. The proband's healthy brother carried none of the variants. According to the American College of Medical Genetics and Genomics guidelines, V1 was classified as likely pathogenic (PVS1, PM2), whereas V2–V4 were considered variants of uncertain significance.

To determine their allelic configuration, we subsequently performed long‐read genome sequencing, which revealed that V2 was in cis with V1, whereas V3 and V4 were in trans with V1 (Figure 1b,c). SpliceAI suggested that V4, located at the exon–intron boundary, had a high likelihood of disrupting splicing (Δscore = 0.96 for donor loss and 0.93 for acceptor loss).

### 2.3 RNA‐Level Consequences of *VPS41* Variants

Because V1 and V4 were prioritized on the basis of phasing and SpliceAI prediction, we investigated their molecular consequences at the RNA level. Transcriptome analysis using RNA extracted from patient‐derived lymphoblastoid cell lines revealed an abnormal splice junction consistent with exon 7 skipping in the patient, whereas control samples showed only canonical exon–exon junctions (Figure S2). A small number of reads also supported exon 15 skipping in the patient (2 reads compared with ~110 reads for the canonical junction), suggesting the presence of a low‐abundance aberrant transcript.

**FIGURE 2:**
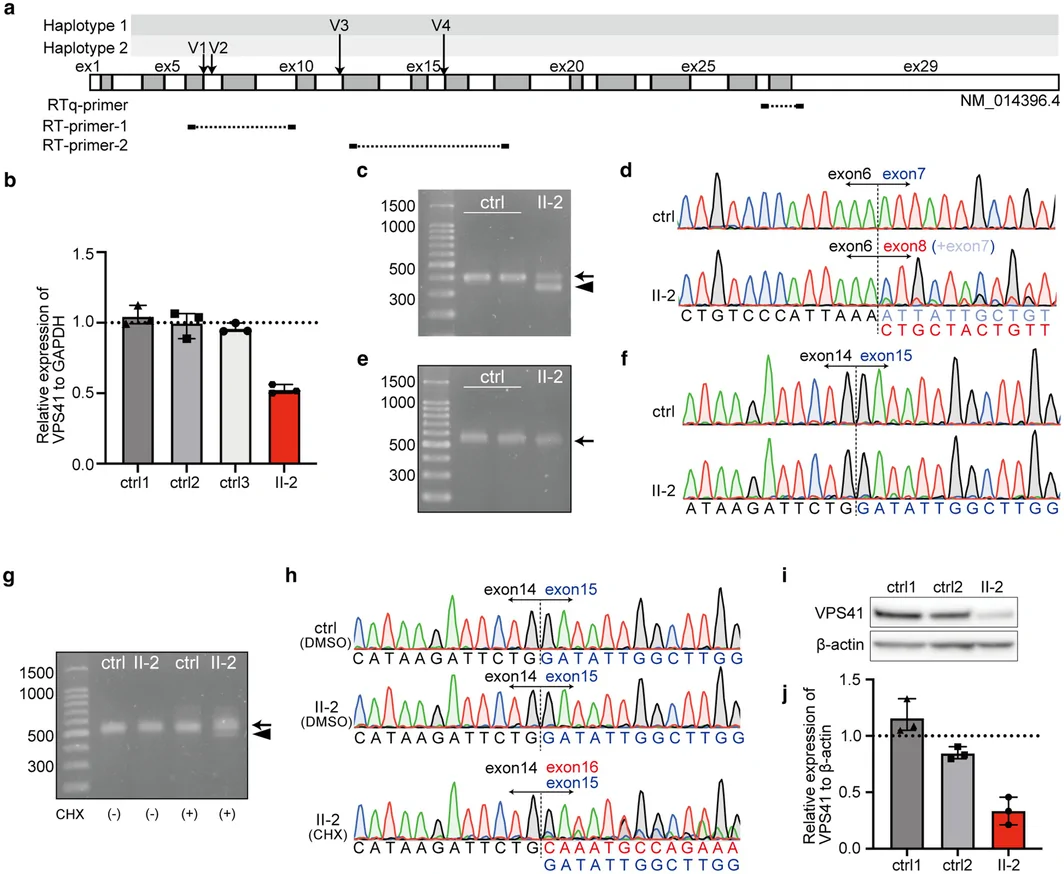
Molecular genetic analysis of the variants. (a) Schematic illustration of *VPS41* mRNA (NM_014396.4) showing the locations of the identified variants, haplotype information, and primer positions used in this study. (b) Quantitative RT–PCR analysis of *VPS41* mRNA expression normalized to that of *GAPDH*. The dotted line indicates the normalized control level (1.0). (c) Representative gel electrophoresis image of RT–PCR using RT–primer‐1. In addition to the expected band (arrow), an additional band was detected (arrowhead). Molecular size markers (bp) are shown on the left. (d) Sanger sequencing of the RT–PCR product amplified with RT‐primer‐1 revealed exon 7 skipping along with normally spliced transcripts. The dotted line marks the exon 6–7 junction. The bases shown below the image represent the observed sequences. (e) Electrophoresis image of RT–PCR using RT–primer‐2. Only the expected band (arrow) was observed, but with reduced intensity. Molecular size markers are shown on the left. (f) Sequencing of the RT–PCR product (RT–primer‐2) confirmed only the reference mRNA sequence. The dotted line marks the exon 14–15 boundary. (g) RT–PCR was performed via RT‐primer‐2 in the presence or absence of cycloheximide (CHX), an indirect nonsense‐mediated decay (NMD) inhibitor. An additional band (arrowhead) appeared with CHX treatment. (h) Sequencing of RT–PCR products from the control (DMSO) and patient (II‐2 with DMSO and CHX) samples. Exon 15 skipping was detected upon CHX treatment. The dotted lines indicate the exon 14–15 junctions; sequences are shown below. (i) Representative Western blot of the VPS41 protein in patient‐derived and control cell lysates. (j) Quantification of the VPS41 protein level normalized to that of β‐Actin. The dotted line represents normalized control levels (1.0). In (b), data are shown for the control (*n* = 3) and one patient sample; in (j), data are shown for the control (*n* = 2) and one patient sample. Each biological sample was measured in technical triplicate, and the mean value was plotted. Because only a single patient‐derived sample was available, no formal statistical testing was performed; the data are presented as individual values together with the means and ranges of the control samples.

We next assessed *VPS41* expression via quantitative RT–PCR and found that *VPS41* mRNA expression was significantly lower in patient‐derived lymphoblastoid cells than in control cells (Figure 2a,b). RT–PCR using two primer sets showed an additional band alongside the canonical transcript with RT‐primer‐1 in the patient sample (Figure 2c). Sanger sequencing revealed that this aberrant band corresponded to exon 7 skipping (Figure 2d). This splicing defect, caused by V1, removes 66 bp but preserves the reading frame, suggesting that the resulting transcript may escape nonsense‐mediated decay (NMD).

In contrast, RT‐primer‐2 detected only the canonical transcript, but with markedly reduced intensity (Figure 2e), and sequencing confirmed no splicing abnormality in the amplified product (Figure 2f). After cycloheximide (CHX) treatment, an additional shorter band became evident and was identified as an exon‐15–skipped transcript lacking 62 bp (Figure 2g,h). This exon 15 skipping introduces a frameshift and premature termination, indicating that V4 induces an out‐of‐frame aberrant transcript subject to NMD.

### 2.4 Protein‐Level Impact of Aberrant *VPS41* Transcripts

To assess the effects of these transcripts at the protein level, we performed Western blot analyses. VPS41 protein was detectable in the patient but was reduced to approximately 30% of the control level (Figure 2i,j). Because monoallelic NMD would be expected to reduce transcript levels by approximately 50%, the greater‐than‐expected reduction suggests that the protein translated from the exon‐7–skipped, NMD‐escaped isoform (V1) may be partially unstable or subject to increased degradation.

### 2.5 Structural Consequences of the Exon‐7–Skipped VPS41 Isoform

To evaluate the structural consequences of the in‐frame exon 7 deletion, we modeled the HOPS complex using AlphaFold and compared the predicted interface properties between the wild‐type complex and the complex containing the VPS41Δex7 isoform (Figure S3). While certain intersubunit interfaces, such as A–E, exhibited increased contact areas and predicted stabilization (ΔΔG = −4.2 kcal/mol), others—including B–E and B–F—showed reduced interface areas and weakened interactions (ΔΔG = 6.6 and 4.3 kcal/mol, respectively; Table S2). These findings suggest that exon 7 skipping alters the interaction landscape of VPS41 within the HOPS complex and may affect overall complex integrity.

### 2.6 Cellular Phenotype Associated With VPS41 Dysfunction

To assess the downstream cellular consequences of VPS41 disruption, we examined patient‐derived lymphoblastoid cell lines using transmission electron microscopy (TEM). TEM revealed characteristic endolysosomal abnormalities, including multiple multivesicular bodies and multilamellar bodies at both 5000× and 10,000× magnification (Figure S4).

### 2.7 Summary of Functional Evidence and Variant Reclassification

Taken together, the RNA, protein, structural, and ultrastructural findings provide functional support for the pathogenicity of both alleles. According to the American College of Medical Genetics and Genomics/Association for Molecular Pathology guidelines and the Clinical Genome Resource Sequence Variant Interpretation recommendations, V1 (NM_014396.4:c.385‐2A>G) was classified as likely pathogenic on the basis of PVS1‐moderate, PM2, and PM3, and V4 (NM_014396.4:c.1247G>A, p.Arg416His) was classified as likely pathogenic on the basis of PVS1 and PM2.

## 3 Discussion

In this report, we present a patient with a VPS41‐related neurodevelopmental disorder caused by compound heterozygous *VPS41* variants. The patient exhibited typical features, including cerebellar ataxia, hypotonia, and ocular motor dysfunction, as well as distinctive craniofacial characteristics (Monfrini et al. 2021; Sanderson et al. 2021; Steel et al. 2020; van der Welle et al. 2021). He also showed previously unreported findings, including swan‐neck deformities and pes cavus. Although pes cavus and hypotonia raised the possibility of peripheral neuropathy, motor nerve conduction studies, performed by a pediatric neurologist at ages 10, 15, and 20 years, consistently showed preserved conduction velocities in both upper and lower extremities, arguing against peripheral neuropathy as the underlying cause of these findings. Spinal cord MRI at age 10 years (C1‐T6) showed no T2 signal abnormalities suggestive of posterior column degeneration in the cervical and upper thoracic cord, although the lumbosacral cord was not imaged. A comprehensive NGS panel for CMT/inherited peripheral neuropathies did not identify pathogenic or likely pathogenic variants in known CMT‐associated genes. While we cannot exclude the possibility that pes cavus represents a coincidental finding in this patient, the available clinical and genetic evidence argues against peripheral neuropathy as its underlying cause. These findings expand the phenotypic spectrum of VPS41‐related disease, although additional cases will be needed to determine whether they represent rare presentations or underrecognized components of the syndrome.

This study also underscores the methodological advantage of long‐read sequencing in rare disease diagnostics. Initial short‐read sequencing identified one likely pathogenic variant together with three variants of uncertain significance, but haplotype phasing could not be resolved because maternal DNA was unavailable. Long‐read sequencing successfully resolved the allelic configuration of the four *VPS41* variants and narrowed the candidates requiring functional evaluation to two, enabling efficient progression to RNA‐based assays and the identification of biallelic likely pathogenic variants.

RNA analysis of patient‐derived cDNA revealed that the c.385‐2A>G splice‐site variant leads to exon 7 skipping, resulting in a 22‐amino‐acid in‐frame deletion (p.Ile129_Lys150del), whereas the c.1247G>A variant causes exon 15 skipping followed by NMD. Exon 7 corresponds to amino acids 129–150 of VPS41 and lies within the N‐terminal region of the protein. Although this region does not fall within the annotated WD40 repeat domain (amino acids ~302–747), it is located near structured domains, including TPR‐like and CHCR motifs, that are predicted to contribute to the scaffold‐like β‐propeller configuration of VPS41 (van der Beek et al. 2024; van der Beek and Klumperman 2025). In‐frame deletion in this region may disturb local folding or interdomain interactions, potentially destabilizing VPS41 or impairing its recruitment into the HOPS complex.

This study has several limitations. First, as a single‐case report, it remains uncertain whether the additional features we observed, such as limb deformities and hearing loss, are specific to VPS41 deficiency or coincidental findings. Second, although long‐read sequencing proved essential for haplotype resolution in this case, its current cost and limited clinical availability may restrict broader application in routine diagnostics. Third, spinal cord MRI was available only from age 10 years (C1‐T6), and updated imaging in adulthood has not been obtainable; this limits our ability to assess spinal cord involvement at later disease stages.

This case expands the phenotypic spectrum of VPS41‐related neurodevelopmental disorders. The additional findings of limb deformities suggest broader phenotypic heterogeneity than previously appreciated.

## 4 Methods

### 4.1 Variant Nomenclature

Variant nomenclature follows HGVS recommendations using the reference transcript NM_014396.4 (NP_055211.2) and genomic build GRCh37.

Detailed descriptions of additional experimental procedures are provided in the Supporting Information. Primer sequences used in this study are listed in Table S3.

### 4.2 Research Ethics Approval: Human Participants

This study involves human participants and was approved by the Committee for Ethical Issues at Nagoya University (protocol numbers: hc22–21 and –22). Written informed consent, including consent for the use of photographs, was obtained from the patient and his legal guardians.

## Author Contributions

Natsuki Nakamura drafted the manuscript and contributed to clinical data collection. Yosuke Nishio and Tomoo Ogi conceived and designed the study. Yosuke Nishio performed genetic data analysis and contributed to manuscript drafting. Tomoo Ogi supervised the study and contributed to manuscript drafting. Hiromi Nyuzuki, Ai Fukushima, Masaki Miura, Yu Kobayashi, Risako Ishioka, Mie Inaba, Jun Tohyama, Takeshi Ikeuchi, and Takeshi Ono contributed to clinical data and critically revised the manuscript. Kotaro Tsukada, Yasuyoshi Oka, Koyo Tsujikawa, Hironobu Morinaga, Yuka Nakazawa, and Shinji Saitoh analyzed genetic data and critically revised the manuscript. All authors approved the final version of the manuscript. Guarantor: Yosuke Nishio is the guarantor of this work and accepts full responsibility for the integrity of the data and the accuracy of the analysis.

## Funding

This study was supported by JSPS KAKENHI Grant Number JP25K19225 (YoN), the Japan Agency for Medical Research and Development (AMED) under Grant Numbers JP24ek0109760 and JP24ek0109617 (Practical Research Project for Rare/Intractable Diseases) (ToO).

## Conflicts of Interest

The authors declare no conflicts of interest.

## Supporting information


**Figure S1:** Clinical features of the patient.
**Figure S2:** Sashimi plot of VPS41 splicing abnormalities detected by transcriptome analysis.
**Figure S3:** Structural prediction and interface analysis of the HOPS complex.
**Figure S4:** Transmission electron microscopy images of lymphoblastoid cell lines.
**Table S1:** Comparison of genetic variants and associated phenotypes between published cases and our patient.
**Table S2:** Predicted inter‐subunit interface properties of the HOPS complex.
**Table S3:** List of primers used in this study.
**Appendix S1:** Detailed clinical information.
**Appendix S2:** Supplementary methods.

## Data Availability

The *VPS41* variant identified in this study has been deposited in ClinVar under the accession IDs VCV004076109.1, VCV004076111.1, VCV004076112.1, and VCV004076110.1 and is publicly available for reference. The other data that support the findings of this study are available upon request.
