# Supplementary material for: Biallelic VPS41 Variants in Autosomal Recessive Spinocerebellar Ataxia 29 Resolved by Long‐Read Sequencing and RNA Analysis
Source: Mol Genet Genomic Med. 2026 Aug 27;14(9):e70285. doi: 10.1002/mgg3.70285 (PMC13519665; doi:10.1002/mgg3.70285)
Supplement: Supplementary file 1 [file MGG3-14-e70285-s001.docx]

**Supporting Information**

*Biallelic VPS41 variants in autosomal recessive spinocerebellar ataxia 29 resolved by long-read sequencing and RNA analysis*

Yosuke Nishio et al. — Molecular Genetics & Genomic Medicine (Manuscript ID 7473969)

**This Supporting Information file contains the following items:**

Appendix S1. Detailed Clinical Information

Appendix S2. Supplementary Methods

Supplementary Figure S1. Clinical features of the patient

Supplementary Figure S2. Sashimi plot of VPS41 splicing abnormalities detected by transcriptome analysis

Supplementary Figure S3. Structural prediction and interface analysis of the HOPS complex

Supplementary Figure S4. Transmission electron microscopy images of lymphoblastoid cell lines

Supplementary Table S1. Comparison of genetic variants and associated phenotypes between published cases and our patient

Supplementary Table S2. Predicted inter-subunit interface properties of the HOPS complex

Supplementary Table S3. List of primers used in this study

**Appendix S1. Detailed Clinical Information**

The patient is a 24-year-old male born at 39 weeks of gestation after an uneventful pregnancy. At birth, his weight was 2905 g (−0.5 SD), length was 48.3 cm (−0.3 SD), and head circumference was 32 cm (−0.9 SD). The patient’s family history was unremarkable, except that his mother died of breast cancer at the age of 35 (Figure S1a). Motor development was within the normal range, with head control at 3 months, sitting at 7 months, crawling at 9 months, pulling to stand at 10 months, and independent walking at 16 months. Cognitive development revealed social smiling at 3 months, first meaningful words at approximately 2 years, and two-word phrases after 3 years, indicating a mild delay.

At 2 years of age, he was referred to a hospital due to gait instability and delayed speech development. Neurological examination revealed truncal ataxia, intention tremor, hypotonia, and the absence of deep tendon reflexes. Between 5 and 7 years of age, ocular motor dysfunction, pes cavus deformity, and bilateral high-frequency hearing loss became apparent (Figure S1b). An intelligence quotient of 49 was recorded by the Wechsler Adult Intelligence Scale-Fourth Edition (WAIS-IV FS) at age 19. At the age of 20, in addition to neurologic findings, dysmorphic facial features, including fullness of the upper eyelids, thick eyebrows, and thick vermilion of the lips, were documented (Figure S1c). Physical examination revealed swan-neck deformities of the second to fourth fingers on both hands, which had gradually progressed since adolescence (Figures S1d and 1e). Importantly, there was no evidence of developmental regression in either the cognitive or motor aspects.

Brain magnetic resonance imaging revealed cerebellar atrophy, predominantly in the anterior region, and poor development of the frontal lobes (Figure S1f). Spinal cord MRI obtained at age 10 years (C1-T6) showed no signal abnormalities in the cervical and upper thoracic cord. Comprehensive neurological evaluations, including blood and cerebrospinal fluid analysis, as well as a muscle biopsy of the right biceps brachii, were inconclusive. Given the presence of hypotonia, areflexia, and pes cavus deformity, peripheral neuropathy was also considered in the differential diagnosis. Nerve conduction studies were performed on three occasions -- at ages 10, 15, and 20 years -- by a pediatric neurologist, with normal results each time, and were interpreted using adult reference values reported in the AANEM Practice Topic.[11] Motor nerve conduction velocities were preserved in both upper and lower extremities across all evaluations. Representative values from the most recent assessment (age 20 years): ulnar motor conduction velocities were 56.4 m/s on the right and 57.7 m/s on the left (reference ≥52 m/s), and tibial motor conduction velocities were 45.8 m/s bilaterally (reference ≥42 m/s). Overall, the nerve conduction findings do not support the presence of a clinically significant peripheral neuropathy.

A comprehensive NGS panel for Charcot-Marie-Tooth disease and inherited peripheral neuropathies (103 genes), genetic testing for spinal muscular atrophy and mitochondrial disorders, and chromosomal microarray analysis failed to detect any definitive pathogenic variants.

**Appendix S2. Supplementary Methods**

**Exome sequencing**

Genomic DNA was extracted from peripheral blood via the QIAamp DNA Blood Midi Kit (QIAGEN) according to the manufacturer’s instructions. Exome capture was performed with the MGIEasy Exome Capture V5 Probe Set (MGI Tech), and sequencing was conducted on a DNBSEQ-T7 platform (MGI Tech) with 150 bp paired-end reads. Reads were aligned to the human reference genome (hg19), and variants with a minor allele frequency >1% in public databases were excluded, except for known pathogenic variants listed in ClinVar or HGMD. We focused on nonsynonymous variants, canonical splice site variants, and small indels. Candidate variants were confirmed via Sanger sequencing via PCR-amplified genomic DNA with custom primers (VPS41-gDNA-1, VPS41-gDNA-2, and VPS41-gDNA-3; sequences are shown in Supplementary Table S3).

**Long-read sequencing**

DNA samples were sheared via the Megaruptor 3 system, and library preparation was performed with the SMRTbell Prep Kit 3.0. HiFi reads were generated on the Revio system and aligned to the GRCh38 reference genome with pbmm2 (v1.10.0). Haplotype-phasing and structural variation calling were conducted via HiPhase (v1.4.2) and pbsv (v2.9.0), respectively.

**RNA extraction and RT‒PCR**

Total RNA was extracted from cells cultured in T25 flasks via the RNeasy Mini Kit (Qiagen) following the manufacturer’s instructions after washing with phosphate-buffered saline (PBS, Wako). Reverse transcription was performed using an equivalent amount of RNA and SuperScript™ IV Reverse Transcriptase (Thermo Fisher). RT‒PCR and quantitative PCR were carried out via the LightCycler 96 System and FastStart Essential DNA Green Master Mix (Roche) with custom primers (RT‒primer, RT‒primer-1 and RT‒primer-2; sequences are shown in Supplementary Table S3).

**Protein extraction and western blotting**

Cells cultured in the T25 flasks were washed with PBS (Wako) and lysed in Tris-based immunoprecipitation buffer (50 mM Tris-HCl, pH 7.4; 0.5% NP-40; and a protease inhibitor cocktail; Roche). Total lysates were separated via e-PAGE on 5%–20% precast gels (E-T/R/D520L, ATTO) and transferred onto polyvinylidene fluoride membranes (Merck Millipore). After being blocked with 5% skim milk in PBS containing 0.1% Tween-20, the membranes were incubated with anti-VPS41 (sc-377271, Santa Cruz Biotechnology) and anti-β-actin (M177-3, MBL) primary antibodies, followed by incubation with horseradish peroxidase-conjugated secondary antibodies (Sigma‒Aldrich). The protein bands were visualized via an Amersham Imager 600 system (Cytiva).

**Transmission electron microscopy**

Samples were fixed in 2% glutaraldehyde and 2% paraformaldehyde in 0.1 M phosphate buffer (pH 7.4), followed by postfixation with 2% osmium tetroxide in the same buffer. The samples were washed, dehydrated through a graded ethanol series, replaced with propylene oxide, and embedded in Quetol 812 epoxy resin. Polymerization was carried out at 60°C for 24 h. Ultrathin sections (90–100 nm) were cut via an ULTRACUT UCT ultramicrotome (Leica) with a diamond knife and stained with 2% uranyl acetate in distilled water for 15 min, followed by the addition of modified Sato’s lead solution for 5 min. The sections were examined via a JEM-1400Plus transmission electron microscope (JEOL).

**Statistical analysis.**

Because this study included only a single affected individual, no formal hypothesis testing was performed for comparisons between the patient and control groups. For quantitative RT‒PCR and immunoblotting, each biological sample was measured in technical triplicate.

The mean value was used for plotting. The data are presented as individual values together with the means and ranges of the control samples. Graphs were generated via GraphPad Prism 10 (GraphPad Software, USA).

**Databases and software**

The following databases were utilized: gnomAD v4.1.0 (https://gnomad.broadinstitute.org/); ToMMo 38KJPN Allele Frequency Panel (https://jmorp.megabank.tohoku.ac.jp/); and HGMD Professional 2024.1 (https://www.hgmd.cf.ac.uk/). For protein structure and interface prediction, AlphaFold 3 (https://alphafoldserver.com/) and PDBePISA (https://www.ebi.ac.uk/pdbe/pisa/) were employed.

**Supplementary Figure S1. Clinical features of the patient.**

(a) Pedigree of the family. The proband is indicated by a filled black symbol marked with “P”; arrows denote individuals evaluated in this study. (b) Right foot showing pes cavus with equinovarus deformity. (c) Facial photographs: frontal view (left) and lateral view (right), demonstrating thick eyebrows, fullness of the upper eyelids, and thick vermilion of the lips. (d) Palmar views of both hands. (e) Lateral view of the right hand showing swan-neck deformities of the fingers. (f) Brain magnetic resonance image showing cerebellar atrophy and poor frontal lobe development (left, midline T2-weighted sagittal image; right, T1-weighted axial image).

**Supplementary Figure S2. Sashimi plot of VPS41 splicing abnormalities detected by transcriptome analysis.**

(a) RNA-seq reads mapped around exon 7 region. The patient’s sample (II-2, red) showed a clear aberrant splice junction connecting exon 6 to 8, corresponding to exon 7 skipping. The control sample (blue) showed only canonical junctions and no aberrant exon 7 skipping.
(b) RNA-seq reads mapped around exon 15 region. A very small number of aberrant junction-spanning reads (2 reads) supporting exon 15 skipping were detected in the patient (II-2, red), while the canonical exon 14 to 15 and exon 15 to 16 junctions remained predominant. The control sample (blue) showed only canonical junctions and no aberrant exon 15 skipping.

**Supplementary Figure S3. Structural prediction and interface analysis of the HOPS complex.**

Predicted structure of the wild-type (left panels) and VPS41Δex7 (right panels) HOPS complex generated by AlphaFold. Each subunit is color-coded as follows: A (pink), VPS41; B (orange), VPS11; C (purple), VPS16; D (gray), VPS18; E (light gray), VPS33A; F (blue), VPS39. Lower panels show schematic representations of inter-subunit interfaces analyzed using ChimeraX. Solid lines represent chain–chain interfaces with relatively large buried solvent-accessible surface areas (SASA), whereas dashed lines represent smaller interfaces (buried SASA < 50% of the largest interface), as defined by the ChimeraX *interfaces* tool.

**Supplementary Figure S4. Transmission electron microscopy images of lymphoblastoid cell lines.**

Upper panels show images at 5,000× magnification; lower panels at 10,000× magnification. Patient-derived cells display multiple multivesicular bodies and multilamellar bodies. Scale bars: 1 μm (upper panels), 0.5 μm (lower panels).

**Supplementary Table S1. Comparison of genetic variants and associated phenotypes between published cases and our patient**

Variants are described according to HGVS nomenclature using reference sequence NM_014396.4 (NP_055211.2).

MRI, magnetic resonance imaging; na, data not available; nd, not documented.

**Supplementary Table S2. Predicted inter-subunit interface properties of the HOPS complex**

Interface properties, including interface area (Å²) and estimated interaction free energy (ΔG, kcal/mol), were assessed using PDBePISA. ΔInterface_area and ΔΔG represent the differences between WT and VPS41Δex7 complexes. Positive ΔΔG values indicate predicted weakening of interactions, whereas negative values indicate stabilization. Interfaces showing substantial loss of interaction area or increased ΔΔG suggest that exon 7 skipping may perturb specific inter-subunit contacts within the HOPS complex, potentially affecting overall complex integrity.

**Supplementary Table S3. List of primers used in this study**
